# Circumcision with the Plastibell Device in Hooded Prepuce or Glanular Hypospadias

**DOI:** 10.1155/2009/864816

**Published:** 2009-10-26

**Authors:** Seyed A. Mousavi, Hamid Mohammadjafari

**Affiliations:** ^1^Department of Pediatric Surgery, Faculty of Medicine, Mazandaran University of Medical Sciences, Sari, Iran; ^2^Department of Pediatric Nephrology, Faculty of Medicine, Mazandaran University of Medical Sciences, Sari, Iran

## Abstract

*Purpose*. To retrospectively review our experience in infants with glanular hypospadias or hooded prepuce without meatal anomaly, who underwent circumcision with the plastibell device. Although circumcision with the plastibell device is well described, there are no reported experiences pertaining to hooded prepuce or glanular hypospadias that have been operated on by this technique. *Materials and Methods*. Between September 2002 and September 2008, 21 children with hooded prepuce (age 1 to 11 months, mean 4.6 months) were referred for hypospadias repair. Four of them did not have meatal anomaly. Their parents accepted this small anomaly and requested circumcision without glanuloplasty. In all cases, the circumcision was corrected by a plastibell device. *Results*. No complications occurred in the circumcised patients, except delayed falling of bell in one case that was removed by a surgeon, after the tenth day. *Conclusion*. Circumcision with the plastibell device is a suitable method for excision of hooded prepuce. It can also be used successfully in infants, who have miniglanular hypospadias, and whose parents accepted this small anomaly.

## 1. Introduction

Circumcision is a very old procedure and has been widely practiced since ancient times [1, 2]. However, it is a routine procedure in Muslim countries such as Iran [3]. At present, in many countries such as the United States, the decision to circumcise is made by the parents and is performed on more than 80% of newborn males [2, 4].

In many references preputial defect is a contraindication for early circumcision [5]. This defect could be a sign of genital anomaly such as hypospadias. Although some people in Iran have named this preputial defect God's circumcision!, They usually prefer to excise remaining prepuce. Most hypospadias repairs performed in the world involve the glanular type [6, 7]. Without chordee, it is only a cosmetic handicap. Occasionally, the wishes of the parents are to be that of a circumcised penis without glanuloplasty.

Plastibell has established itself as an acceptable form of circumcision and is the most frequently used device in the world. Popularity of the device can be ascribed to its claimed ease of use [8]. Although circumcision with the plastibell device is well described, there are no reported experiences pertaining to hooded prepuce or glanular hypospadias that have been operated on by this technique. We reviewed the outcome of infants with glanular hypospadias or hooded foreskin who underwent circumcision with plastibell device.

## 2. Materials and Methods

Between September 2002 and September 2008, 184 children (mean age 3 years, range 2 months to 15 years) were referred for hypospadias repair. 43 patients had a glanular type and 17 parents preferred to do circumcision without hypospadias repair, while the remaining 26 patients were corrected using a magpi or TIP. We had four infants who had hooded prepuce without hypospadias. Therefore, 21 infants with preputial defect were circumcised by using the plastibell device. (Aged 1 to 11 months, mean 4.6 months) we did not detect any significant chordee by physical examination so it was not assessed in the post operative period.

The study was approved by the Ethics Committee of Mazandaran University of Medical Sciences. All participants were healthy males without any medical indication. Cases in which the foreskin was required for chordee release and those in which the meatus was steno tic and unacceptable for parents, were excluded from circumcision. These were operated on by one pediatric surgeon. After the primary evaluation and explanation to parents, the operation was performed.

**Figure 1:**
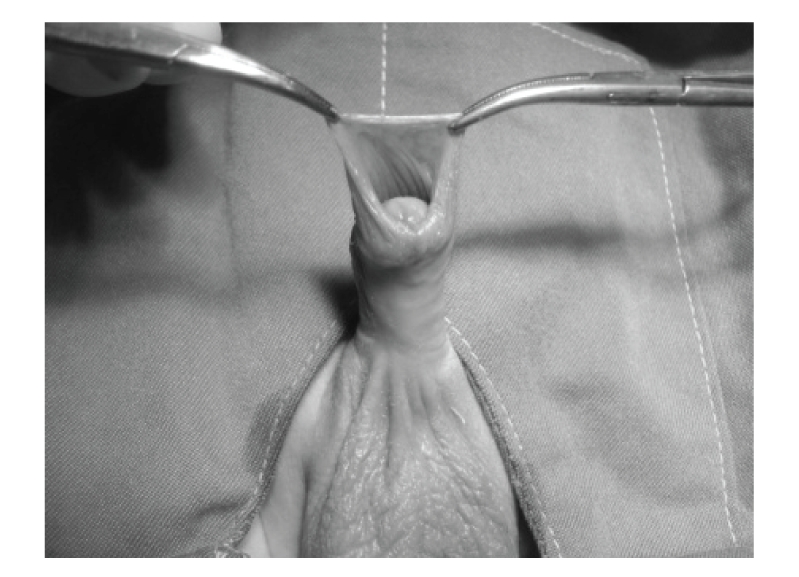
Hooded prepuce.

**Figure 2:**
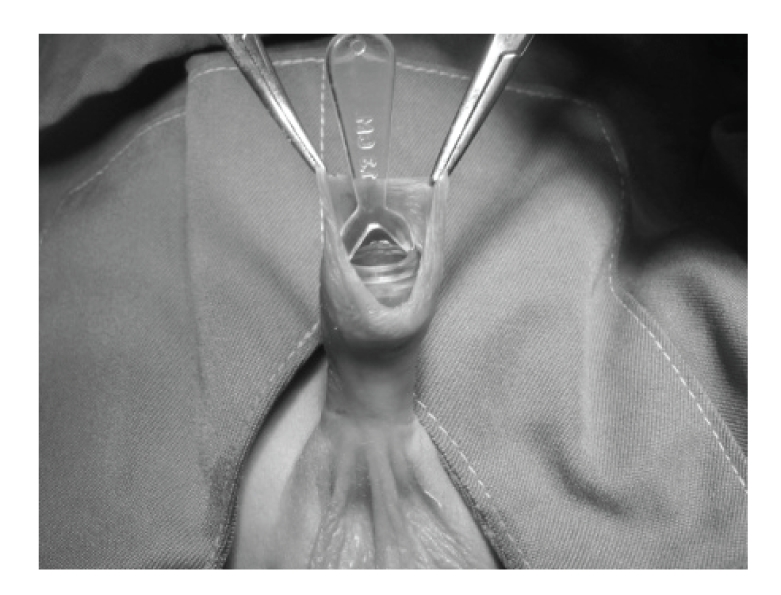
Putting the adequate bell on the glans.

**Figure 3:**
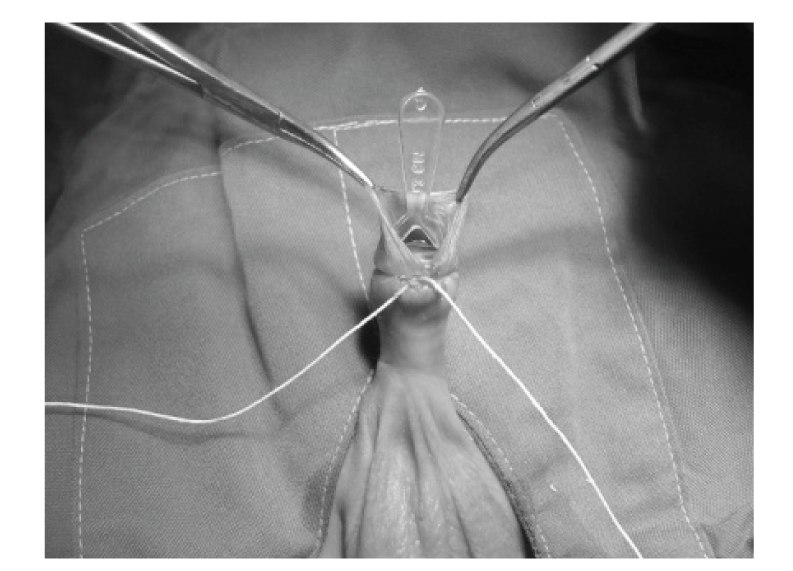
Tying the foreskin over the bell.

**Figure 4:**
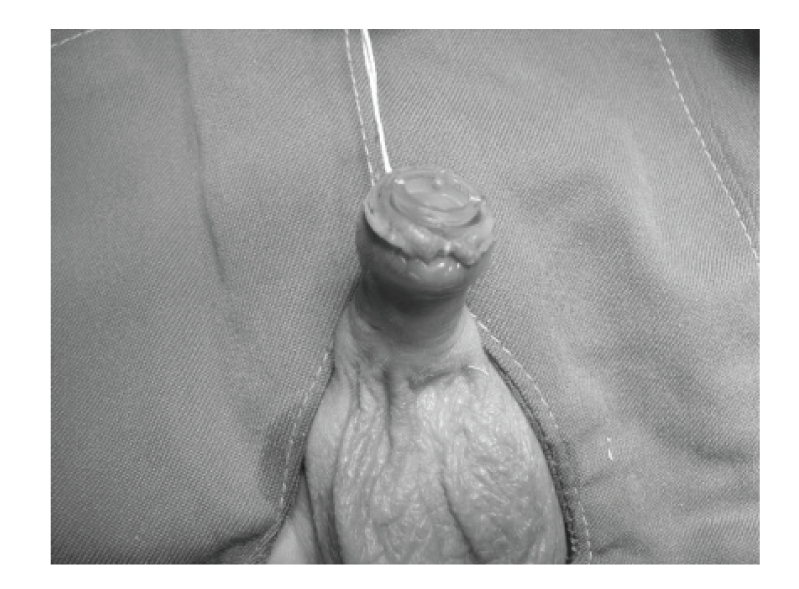
Cutting the prepuce.

**Figure 5:**
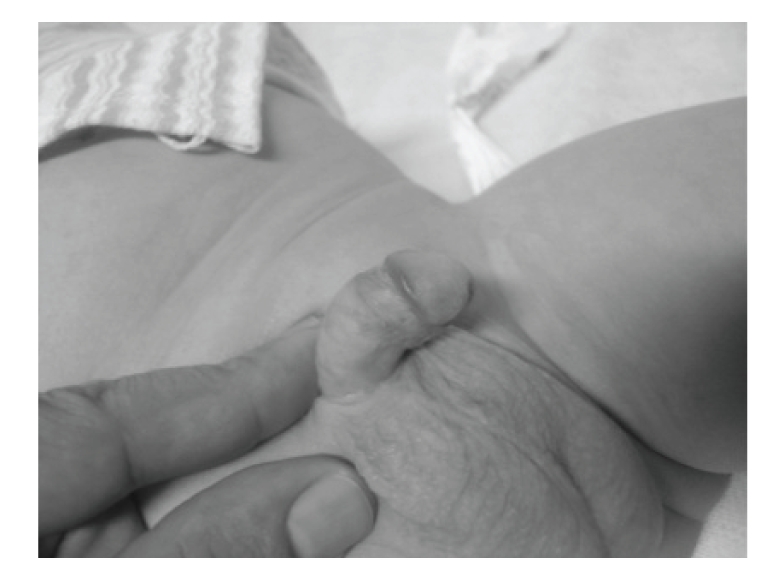
Post operative view after one month.

Infants were not fed for 1-2 hours prior to the procedure. After placing an infant on a circumcision restraint board, the skin was prepared with povidone iodine (10%) solution. A dorsal nerve block was administered using 0.2 mL/kg of 2% lidocaine, with a 27-gauge needle. The location of the coronal sulcus on the shaft skin is marked. Because the preputial ring is open, it does not need to be crushed and incised the foreskin for placement of the bell (Figure 1). Therefore, a blunt probe easily lyses the adhesions between the glands and foreskin to clean the smegma. Then, the correct size of plastibell is selected (Figure 2). The bottom edge of the bell should completely cover the corona. The dorsal and lateral sides of the foreskin is pulled over the bell until the previously marked level of the coronal sulcus lies over the groove in the bell and ventral side locate as near as possible to groove. A suture is tied around the foreskin over the tying groove in the plastibell. The size of ventral foreskin defect is different in patients. If there is not any skin we have to tie only over the bell and in others the minimal ventral skin could be involved (Figure 3). The foreskin is excised just past the outermost groove, with care taken not to injure the glands (Figure 4). A minimal dressing of antibiotic ointment (gentamicin) and gauze was placed with the expectation of it falling off spontaneously. An acetaminophen drop was used as an analgesic. The bell would eventually fall off, after necrosis within several days. The parents of subjects were informed to return to the clinic, if the time of bell separation exceeded more than 10 days. In addition, parents were directed to do sits bath with soapy water twice per day and also apply a liberal amount of ophthalmic ointment gentamicin to the operative site until such time of the bell falling off. All children were followed up, until the wound was fully healed (Figure 5). Operative time was recorded and pos-operative complications, outcomes, parents' satisfaction were assessed. Infection, bleeding or hematoma, excess mucosa, bell disposition (entrapping the ring) and delayed falling were considered as complications.

## 3. Results

The mean followup after surgery was two months. No serious complications were indicated in our series.

The only complication was delayed separation of ring in an eight month old infant whose bell did not separate after the 10th day; therefore, we removed the cup accordingly by cutting the tie. Thus, the overall complication rate was 4.7%. All infants voided spontaneously after surgery and none developed urinary retention needing catheterization. The average procedure time (in spite of the time needed for anesthesia) was 4 minutes.

Eventually, the cosmetic results were excellent in all children and there were no complaints by infants parents regarding the cosmetic aspect.

## 4. Discussion

Circumcision is a very old procedure and complications are considered rare and usually trivial [8]. In many references, preputial defect is a contraindication for early circumcision [5], as it could be a sign of genital anomaly such as hypospadias. On the other hand, the most hypospadias repairs performed in the world involve the glanular type [6, 7]. All procedures for repair of this anomaly have been more cost effective and complication rate is reported to be higher than common circumcision.

The technique of choice for circumcision in infants with preputial defect remains uncertain and usually is performed by conventional dissection technique. The treatment of anterior hypospsdias depends on the cultural preference of the child's family. Many patients with anterior hypospsdias do not have a functional defect, lacking significant penile curvature, and will be able to void with a straight stream. Therefore, the goal of placing the meatus in its normal position within the glands is essentially cosmetic. Occasionally, the wishes of the parents are to be that of a circumcised penis without glanuloplasty. We have reviewed the outcome of infants with glanular hypospadias who received the circumcision with the plastibell device. The plastibell technique was based on the usual manner, with minor modification.

Routine neonatal circumcisions can be a safe procedure [9]. The overall complication rate of the procedure is between 0.19% and 3.1% [10]. Although many studies for circumcision with plastibell device have been preformed, however, there is no report determining this technique may be useful in children with hooded prepuce [11–13]. These studies propose that circumcision with PD in healthy prepuce is a simple method and complications including hemorrhage, local infection, sepsis, metal ulceration, and poor cosmetic results are rare.

In the present series, we had 21 infants with glanular hypospadias or hooded prepuce in which the circumcision was performed by plastibell. This was a new experience and despite the hooded prepuce, and the incomplete coverage of bell by foreskin, this technique had excellent results with no serious complications. We had one case whose bell was separated by surgeon after ten days due to delayed falling of bell. The cosmetic and functional results were excellent in all infants and no parents complained about the cosmetic aspect.

 However, there is at least a theoretical risk of urethral injury if the ventral shaft skin is manipulated, as the corpus spongiosum is not well developed, and the urethra may be close to the skin. So it is clear that a PD on a child with mild hypospadias/preputial defect is only safe if chordee has been ruled out.

Although the number of our cases was limited, the complication rate is similar to our previous study in normal prepuce and also, the preputial defect did not worsen the success rate of the procedure [3].

As reported in other studies [3, 14], an obvious advantage of using the Plastibell was the brief surgery time (4 minutes). In our series, the average procedure time was similar to operation in infants with intact prepuce.

The main limitation of our study is due to the few number of cases. This study assessed only 21 infants. Therefore, we are planning to followup with more subjects in terms of possible complications. Larger prospective studies are needed to ascertain similarity in outcome.

In conclusion, using the plastibell device for circumcision is a suitable method for treating the infants with mini glanular hypospadias who their parents prefer circumcision without glanuloplasty. It can also be used successfully in infants with normal meatus, who do not have an intact prepuce. It is simple, fast with an acceptable cosmetic result and few complications. It is economical and can be performed under local anesthetic as an out-patient. This operation is preformed when the child is of any age, even a neonate. It should be noted that as the meatus is not manipulated, it gives a good cosmetic result, with no meatal stenosis, which is important in glanuloplasty.

 Our study showed that circumcision with the plastibell device in these children is successful. We would recommend circumcision by this technique in infants with glanular hypospadias, if parents would prefer their son to be circumcised without glanuloplasty.

## References

[1] Untley JS, Bourne MC, Munro FD, Wilson-Storey D (2003). Troubles with the foreskin: one hundred consecutive referrals to pediatric surgeons. *Journal of the Royal Society of Medicine*.

[2] Lafferty PM, MacGregor FB, Scobie WG (1991). Management of foreskin problems. *Archives of Disease in Childhood*.

[3] Mousavi SA, Salehifar E Circumcision complications associated with the Plastibell device and conventional dissection surgery: a trial of 586 infants of ages up to 12 months.

[4] Goodman MT, Hernandez BY, Shvetsov YB (2007). Demographic and pathologic differences in the incidence of invasive penile cancer in the United States, 1995–2003. *Cancer Epidemiology Biomarkers and Prevention*.

[5] Davenport M (1996). ABC of General Surgery in Children: problems with the penis and prepuce. *British Medical Journal*.

[6] Wallon P, Saint-Supery G, Bucco P (1984). Plastic surgery to the prepuce, known as the Lille operation, for treatment of distal hypospadias. A report on 138 cases. *Chirurgie Pediatrique*.

[7] Snodgrass WT, Koyle MA, Baskin LS, Caldamone AA (2006). Foreskin preservation in penile surgery. *Journal of Urology*.

[8] Sörensen SM, Sörensen MR (1988). Circumcision with the plastibell device a long-term follow-up. *International Urology and Nephrology*.

[9] Lazarus J, Alexander A, Rode H (2007). Circumcision complications associated with the Plastibell device. *South African Medical Journal*.

[10] Okeke LI, Asinobi AA, Ikuerowo OS (2006). Epidemiology of complications of male circumcision in Ibadan, Nigeria. *BMC Urology*.

[11] Peng Y-F, Cheng Y, Wang G-Y (2008). Clinical application of a new device for minimally invasive circumcision. *Asian Journal of Andrology*.

[12] Holman JR, Lewis EL, Ringler RL (1995). Neonatal circumcision techniques. *American Family Physician*.

[13] Gee WF, Ansell JS (1976). Neonatal circumcision: a ten-year overview: with comparison of the Gomco clamp and the Plastibell device. *Pediatrics*.

[14] Fraser IA, Allen MJ, Bagshaw PF, Johnstone M (1981). A randomized trial to assess childhood circumcision with the Plastibell device compared to a conventional dissection technique. *British Journal of Surgery*.

